# Authors’ reply

**DOI:** 10.1192/bjb.2020.15

**Published:** 2020-04

**Authors:** Rachel Gibbons, Fiona Brand, Anne Carbonnier, Alison Croft, Karen Lascelles, Gislene Wolfart, Keith Hawton

**Affiliations:** Consultant Psychiatrist, Priory Group, email: rachelgibbons@nhs.net; Psychiatric Nurse, Oxford Health NHS Foundation Trust; Consultant Child and Adolescent Psychiatrist, South Oxfordshire Child and Adult Mental Health Services (CAMHS) and Medical Lead for South Oxon CAMHS; Consultant Clinical Psychologist with the Medic Support Service, Oxfordshire Psychological Services and Oxford Cognitive Therapy Centre, Oxford Health NHS Foundation Trust; Nurse Consultant in Suicide Prevention, Oxford Health NHS Foundation Trust; Lead Psychologist, Oxford Mind and Body; Director, Centre for Suicide Research, University of Oxford and Consultant Psychiatrist, Oxford Health NHS Foundation Trust, Warneford Hospital

**Authors’ reply:** We are grateful for Professor Morgan's engagement with our paper,^1^ and that we have stimulated debate in this important area. We do, however, feel it necessary to respond to two fundamental misinterpretations of our discussion about the predictability and preventability of suicide.

First, the letter states that we were asserting there is ‘no evidence that suicide risk assessment in clinical practice can usefully guide clinical decision-making’, whereas we actually said that our ability to predict patients at the highest level of risk is limited and that despite the ubiquity of advice to use suicide risk assessment in clinical practice, the positive predictive value is low and there is no evidence that these assessments can usefully guide decision-making.

Second, Prof. Morgan asserts that we were advocating ‘ignoring the value of suicide ideation’, which was not indicated at any point in the paper.

Suicidal thoughts are very common; however, completed suicide is rare. The process that leads any individual to take their life is often poorly understood. Given this, identifying the specific individuals most likely to die by suicide is a very a challenging task. An overly high personal and systemic expectation of a clinician's capacity to predict suicide in any individual case is unreasonable and can lead to increased feelings of distress and blame following a death. The distorted focus on psychiatrists’ role to prevent suicide obfuscates their important role in working to alleviate mental pain, encourage recovery and improve quality of life.

We strongly advocate dynamic formulation of risk as part of clinical care but question an over-reliance on risk assessment tools that have little or no face validity. There is now a general consensus^3,4,5^ that these tools do not predict likelihood of suicide. Indeed, the National Institute for Health and Care Excellence guideline on self-harm from 2011, based on reviewing evidence, states: ‘Do not use risk assessment tools and scales to predict future suicide or repetition of self-harm’, nor ‘to determine who should and should not be offered treatment or who should be discharged’.^6^

We agree with Prof. Morgan that there is much to affirm the important role of assessing suicidal ideation in guiding clinical decision-making. Suicidal ideation is important not only as an indicator of potential suicide but also as a clear sign of mental distress. Self-destructive thoughts are a key symptom in various types of mental disorder, including depression and personality disorders. These thoughts need to be assessed in the context of other factors, as recommended by various agencies, including the National Confidential Inquiry into Suicide and Homicide,^7^ such as previous self-harm, social circumstances, patients’ relationship with their mental health team, access to means and early follow-up. We also endorse the current move away from primarily focusing on trying to identify patients at most risk to individualised safety planning for all patients.

We thank Dr Calcia for her supportive letter.^8^ We particularly agree with her focus on the need to prepare trainees for the experience of a patient death by suicide. In this respect, we include the following quotes from psychiatrists in our survey.

‘Start training the trainees early on suicide and suicide prevention. Do not neglect the impact it will have on level of functioning and on career choices. Building resilience is essential to help doctors sustain the possibly repeated events of patient suicide.’

‘I think the training on this subject should be part of core and higher training and higher trainees should have some mentoring and exposure to this process.’

While the above comments may seem counter to the current focus on a zero suicide policy, in our opinion they do reflect the reality of psychiatric practice. Preparation for this often painful event is likely to decrease personal trauma and reduce the risk of losing valuable staff from our profession.
